# Protocol for evaluation of iTEST, a novel blended intervention to enhance introspective accuracy in psychotic disorders

**DOI:** 10.1038/s44277-024-00024-7

**Published:** 2025-02-14

**Authors:** Sarah A. Berretta, Nicole Abaya, Emma Parrish, Lauren E. McBride, Raeanne C. Moore, Robert Ackerman, Philip D. Harvey, Amy E. Pinkham, Colin A. Depp

**Affiliations:** 1 School of Behavioral and Brain Sciences, The University of Texas at Dallas, Richardson, TX USA; 2 Department of Psychiatry, University of California San Diego, La Jolla, CA USA; 3 San Diego State University/University of California San Diego Joint Doctoral Program in Clinical Psychology, San Diego, CA USA; 4 Research Service, Bruce W Cater Miami VA Healthcare System, Miami, FL USA; 5 University of Miami Miller School of Medicine, Miami, FL USA

**Keywords:** Clinical trials, Therapeutics

## Abstract

Poor introspective accuracy (IA), defined as inaccurate judgments of one’s abilities and performance, is a strong and independent predictor of functional impairment in people with psychotic disorders. However, there are currently no treatments that directly target IA in this population as a primary outcome. We describe a protocol for a clinical trial to test a newly developed blended digital intervention, Improving Thinking through Everyday SelfAssessment Training (iTEST), aimed at improving IA in people with psychotic disorders to improve functional outcomes. iTEST involves daily training consisting of feedback on IA in mobile cognitive tests, coupled with individual coaching that applies improved IA to participant-identified recovery goals. Following the NIMH experimental therapeutics paradigm, the first step in the evaluation of iTEST is an open trial in 60 individuals with psychotic disorders to assess 1) feasibility and acceptability, and 2) whether the intervention leads to clinically significant improvement in an objective target: IA on trained tasks along with transfer to an untrained task-based measure of IA. After programming of the mobile intervention and the creation of treatment manuals, enrollment for an open trial started in November 2023 and will be completed by April 2025. If effective, iTEST could be integrated with cognitive training and other rehabilitative interventions to boost the impact on functional outcomes. Trial registration: ClinicalTrials.gov NCT05899348.

## Lay summary

Introspective accuracy is the ability to assess one’s own skills and performance. Impairment in introspective accuracy is common among people with psychotic disorders, and poor introspective accuracy is linked to worse daily functioning. There are currently no interventions that directly target introspective accuracy. In this manuscript, we present a study protocol for a digital health intervention aimed at improving introspective accuracy among people with psychotic disorders.

## Introduction

Psychotic disorders (i.e., schizophrenia and schizoaffective disorder) affect approximately 1.5% of the population and cause the most substantial disability of any psychiatric disorder [1–3]. A growing body of literature spanning cognitive science, educational research, and more recently, mental health, links inaccurate judgments of performance, or poor Introspective Accuracy (IA), with diminished real-world functioning in psychotic disorders [4–8]. IA is the discrepancy between objective performance and subjective estimation of performance [5, 7]. IA impairments span psychiatric illnesses, yet aberrant IA appears to be more common and pronounced in psychotic disorders [6, 9] and most frequently manifests as overconfidence in abilities [10]. However, there are no interventions that directly target IA. Here, we present a protocol for the initial phase of a new blended digital health intervention called Improving Thinking through Everyday Self-Assessment Training (iTEST) that aims to improve IA among people with psychotic disorders.

Treatments targeting IA are limited. Existing interventions to date target decision-making biases associated with psychotic symptoms, with other outcomes being considered secondary [11, 12]. IA, as defined in this protocol, is distinct from other domains of self-awareness such as clinical insight, which focuses on beliefs about the illness and its treatment, and metacognition, which emphasizes reducing cognitive errors like jumping to conclusions. Unlike these areas, IA directly addresses the personalized evaluation of one’s own skills and abilities and is measured objectively through the discrepancy between subjective estimates and actual performance. iTEST is the first intervention to specifically target IA in populations with psychotic disorders. Prior research suggests that IA is malleable and that IA skills can be transferred across domains [13, 14]. For example, an experiment in healthy adults improved IA on a trained task involving repeated judgments of correctness and led to the transfer of training to a novel task [15]. Education research among healthy adults indicates that interventions targeting improvement in task-based judgments of performance lead to improvements in learning [16, 17]. Other studies have found that improvements in task-based judgments extend to functional pursuits, such as employment searches [18, 19].

The iTEST intervention represents an attempt to translate basic science findings to clinical use. iTEST is a blended intervention, coupling automated mobile, remotely delivered task-based training in IA with personalized coaching in applying improved IA to everyday behaviors. The task-based components of iTEST are built upon previously validated mobile cognitive tasks that target verbal learning and facial emotion recognition, including the Mobile Variable Difficulty List Memory Test (VLMT) [20] and the Mobile Electronic Test of Emotional Recognition (METER) [21]. These tasks were selected for iTEST because studies have shown that average performance remains stable over time, they correlate with in-lab performance on tests of the same construct in people with psychotic disorders, and are associated with functional outcomes [22]. The verbal memory and facial emotion recognition domains were specifically chosen because they represent core cognitive functions that are frequently impaired in psychotic disorders and have direct implications for daily functioning. Memory deficits significantly impact occupational and social outcomes [23], while emotion recognition abilities are fundamental to social cognition and interpersonal relationships [24]. Additionally, performance on these tasks has been linked to motivation levels, which is a key factor in functional recovery [25]. Finally, we have validated these two tasks in mobile cognitive test formats as compared to in-lab measures in the study population, thus providing a basis from which to develop iTEST [26, 27]. IA judgments of performance in relation to actual performance are captured after the completion of each task, with increasing levels of difficulty as IA skills improve. The focus of coaching in iTEST is to support the translation of improved IA to functional goals that are meaningful to participants. Coaching is intended to also support participants in using compensatory strategies to achieve their goals and applying balanced thinking in approaching goal steps.

The evaluation of iTEST follows the NIMH’s Experimental Therapeutics paradigm. Supported by the NIMH’s two-phase R61/R33 grant mechanism, the first phase of iTEST (R61) is an open trial to evaluate the intervention’s feasibility, acceptability, and impact on IA. Here, impact is defined by IA improvements on both trained and untrained tasks as an indicator of generalization. Because iTEST is novel, a secondary aim of the R61 phase is to establish an optimal “dose” of the intervention by evaluating impacts at 8-, 12-, and 16-weeks post-baseline and selecting the shortest duration at which improvements are seen.

## Materials and Methods

### Setting and regulatory oversight

Participants are recruited from two study sites: the University of California San Diego (UCSD) and The University of Texas at Dallas (UTD). Recruitment settings include medical centers, public mental health clinics, local community clinics, and non-profit organizations. Methods include the use of flyers, word of mouth strategies, clinician referrals, and online advertisements. The University of California San Diego (UCSD) Institutional Review Board (IRB) approved the clinical trial, and the two-site study employs a SMART IRB [28] wherein the University of Texas at Dallas (UTD) is reliant on UCSD’s IRB. Each participant will complete the University of California San Diego Brief Assessment of Capacity to Consent to confirm decisional capacity before providing written informed consent [29]. The trial is registered in ClincialTrials.gov as NCT05899348.

### Participant eligibility

***Inclusion criteria*** for this study include: (1) voluntary informed consent to participate and capacity to consent; (2) aged 18 to 65; 3) DSM-5 diagnosis of schizophrenia or schizoaffective disorder based on the Structured Clinical Interview for DSM Disorders-5 (SCID-5; First et al., 2015) and available medical record review; (4) ≥6th grade reading level on the Wide Range Achievement Test-4 (WRAT-4) Reading subtest [30]; (5) no hospitalizations or medication class changes in two months prior to enrollment, determined by best-estimate history with information from medical records; (6) availability of a clinician with at least monthly contact (staff member, case manager, other mental health clinician) OR close associate (family member, friend) with at least biweekly contact who can serve as an informant.

***Exclusion criteria*** are: (1) greater than moderate disorganization on the PANSS (P2-Disorganization item >5) due to demonstrated difficulties with adherence and engagement with blended interventions [31]; (2) DSM-5 moderate or severe substance use disorder in past three months based on the SCID-5; (3) level of care required that interferes with outpatient therapy (e.g., hospitalized; severe medical illness); (4) unable to interact with a smartphone (e.g., due to visual impairment); (5) lack of functional impairment, which is operationalized as having full-time employment and/or financial responsibility for their household.

***Rationale for inclusion/exclusion criteria*:** We considered a broader transdiagnostic focus, but our preliminary data on direct comparisons strongly suggests that people with psychotic disorders have significantly more profound deficits in IA [32], notably in responsiveness to feedback [33], than do people with bipolar disorder. We considered screening for impairment in IA; however, screening for IA is not currently clinically translatable.

### Timeline

**Fig. 1:**
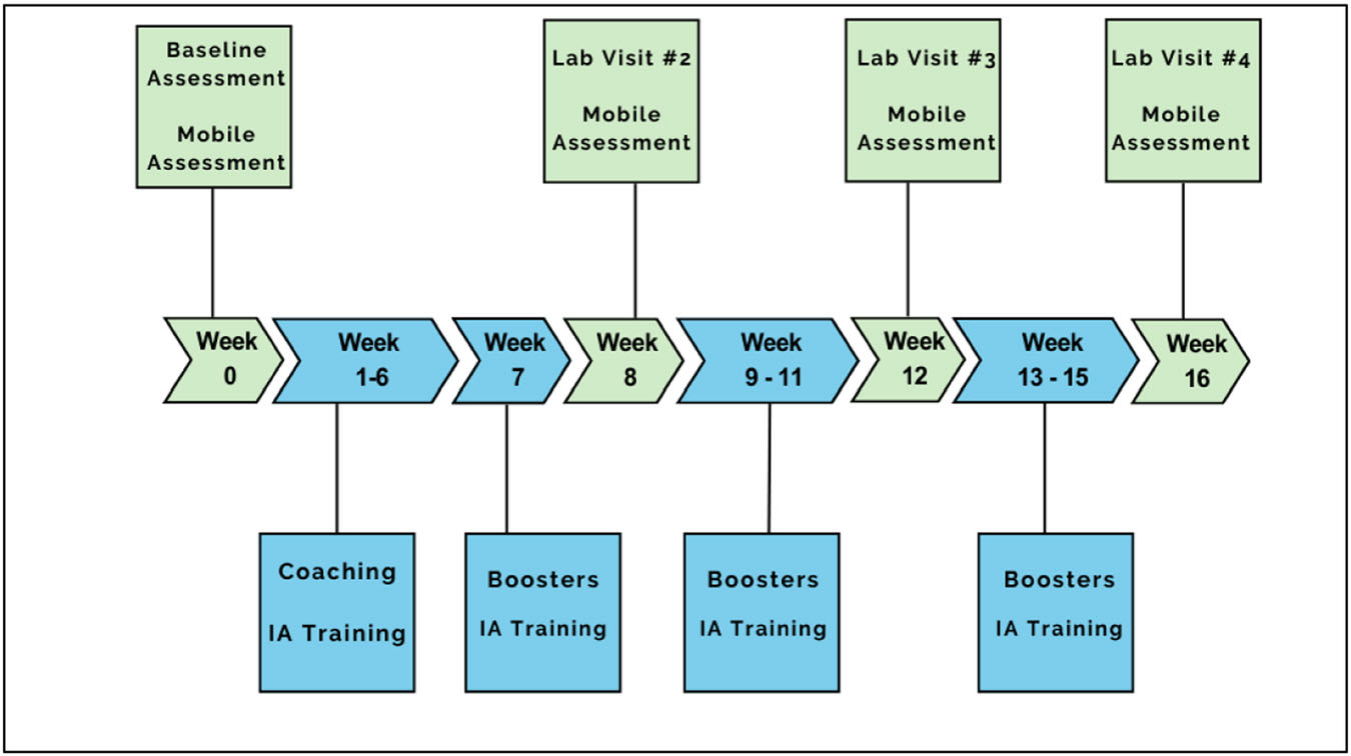
Participation Timeline.

Figure 1 depicts the timeline for a participant in the study. After eligibility screening, participants complete a baseline assessment (see Measures below). Following the baseline visit, participants meet their coach and receive a tutorial regarding iTEST mobile procedures. They then complete a 6-day baseline assessment of IA using the mobile procedures before beginning the 16-week training program outlined below. During weeks 1–6, participants meet weekly with their coach for 60 minutes and complete mobile training, which includes IA feedback, 6 days per week. Subsequent training weeks [8–16] also include mobile procedures 6 days per week with feedback provided and bi-weekly 15-minute phone check-ins with their coach. Interspersed, there are 3 follow-up in-lab assessment visits and mobile assessments, as with the baseline visit.

### Intervention overview

iTEST was designed to contain the 4 elements linked with increased efficacy in cognitive remediation: trained coaching, repeated cognitive tasks, coaching in cognitive strategies, and a focus on the translation of skills to everyday functional tasks [34]. For cognitive tasks, participants complete word memorization (VLMT) and facial expression recognition (METER) tasks on smartphones 6 days a week, with each session lasting 10–15 minutes. At the conclusion of each task, participants are asked how many of the items they believe they got correct, and these estimations are compared to their actual score to index IA. Our web-based smartphone-compatible software is built around our previously validated NeuroUX’s VLMT and METER tasks [21, 35]. Participants have 6 hours to initiate their daily training and will receive 2 reminders throughout the 6-hour window to prompt them to complete the tasks. If they miss 2 consecutive days, study staff will contact participants for problem-solving and motivational support.

The training task stimuli are sourced from a large normed corpus to ensure no repetition over the trial. Training is designed to support incremental improvement in IA and provides more information and support in the initial phases based on best practices in cognitive remediation programs [36]. Training consists of 3 levels of difficulty with 5 sub-levels (e.g., 1-1, 1-2, etc., through 3-5) that progressively require more competency from the participants. Both the VMLT and METER tasks are used at all levels, and difficulty is manipulated by increasing the number of stimuli. At each level, training includes 3 scaffolding steps. The first is task awareness and orientation, where participants learn to correctly identify the number of stimuli (words or faces) presented. The second scaffolding step involves trial-by-trial feedback. Specifically, participants receive trial-by-trial feedback on the correctness of their responses to inform their overall estimate of the number correct. The third scaffolding step directly addresses IA by asking participants to estimate how many questions they answered correctly. These global performance estimates are compared to actual performance to yield an IA value.

As participants progress through sub-levels of training and demonstrate increased competency, these scaffolding steps are systematically removed. Sub-level 1 includes questions and feedback regarding a number of stimuli, item-by-item correctness, and feedback on IA (e.g., *you said that you got 4 answers correct, but you got 5 answers correct)*. Sub-level 2 drops the question about a number of stimuli and only provides item-by-item feedback and feedback on IA. In sub-level 3, participants are asked to evaluate item-by-item correctness on their own instead of this information being automatically provided. Sub-level 4 asks participants to evaluate their item-by-item correctness but provides no feedback for these individual judgments. In sub-level 5, participants only make an overall IA judgment at the end of the task.

Advancing from each sub-level requires participants to correctly identify the number of stimuli for 3 consecutive days. Advancing difficulty levels requires 3 consecutive days of IA estimates that are ±1 or 0, indicating accurate IA. Participants complete all sub-levels for each level of difficulty.

**Fig. 2:**
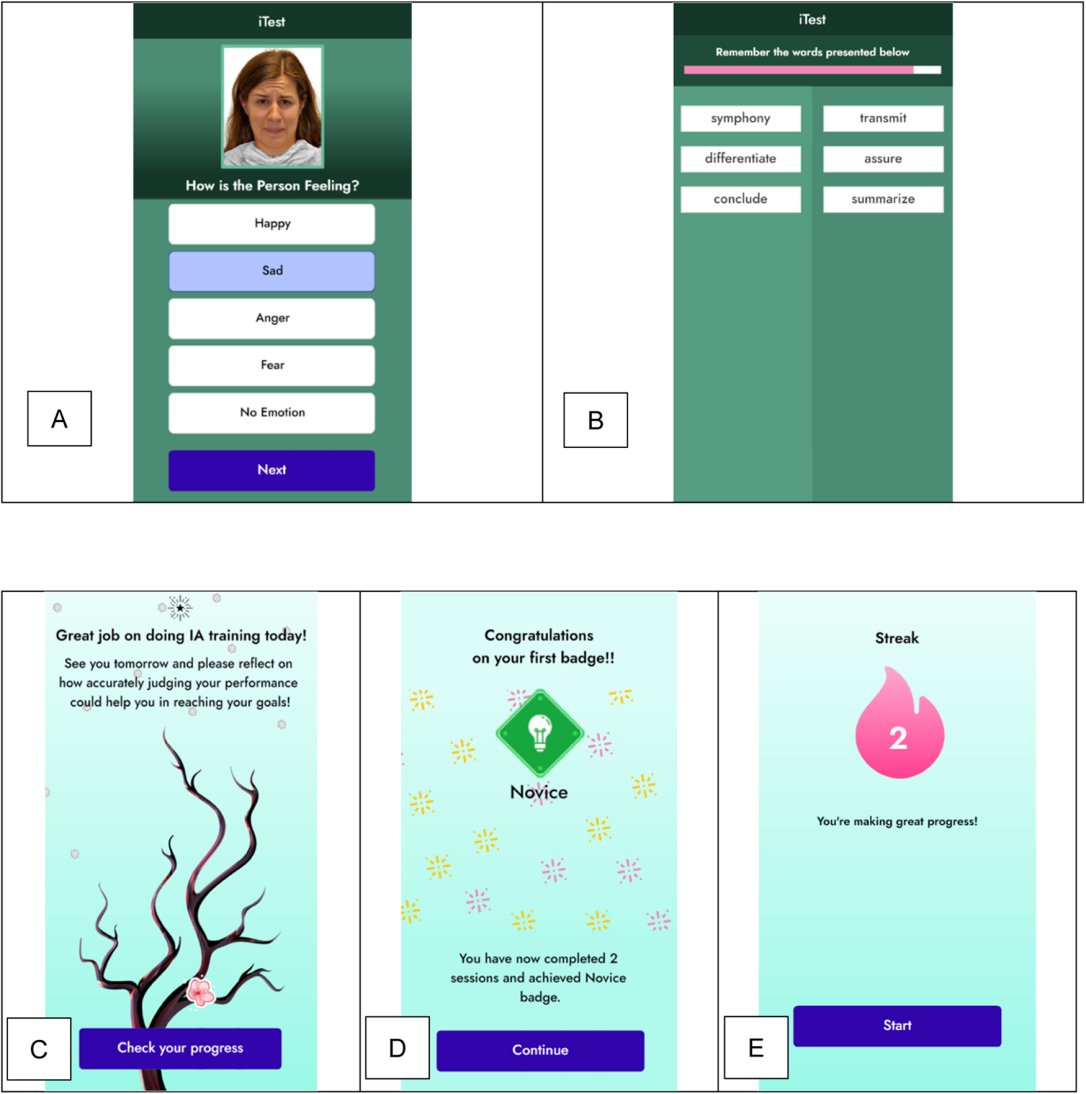
Training task and user engagement screenshots. **A** Stimuli for the METER Social Cognition Task; **B** Stimuli for the VLMT Verbal Learning Task; **C** Tree Symbolizing Progress; **D** Badges Earned for Engagement; **E** Streaks Accumulated through Serial Engagement.

***Design elements to sustain engagement***: Engagement and motivation are encouraged through rewards, feedback, and gamification elements within iTEST [37]. Encouragement to continue training is directly provided in the app; the home screen shows a cherry blossom tree that grows flowers after each training session is successfully completed (see Fig. 2). Streaks are utilized for motivation to complete the daily mobile tasks. Participants can monitor their progress on the application’s dashboard. Additionally, coaches can view a dashboard that displays progress, which is reviewed with the participants in coaching sessions. Each of these design elements, particularly coach involvement, is linked to better adherence in meta-analyses of mobile interventions [38].

### Coaching

iTEST mobile training is combined with weekly 60-minute coaching sessions in weeks 1-6. In weeks 8-16, the same coach provides biweekly 15-minute phone call check-ins. The 60-minute sessions are delivered either remotely or in-person per the preference of the participant. Sessions are manualized (see *Fidelity* below), consistent with our prior blended health interventions [39–42]. The introductory session provides the basis of iTEST to participants and includes psychoeducation on IA, a review of the participant’s baseline iTEST scores, a discussion of compensatory strategies in goal-setting, and a personalized goal identification activity [42, 43]. Thereafter, each iTEST coaching sessions involves: (1) review and feedback on iTEST adherence and IA scores; (2) psychoeducation on the impact of inaccurate IA on everyday activities; and (3) collaborative training, selection, and application of IA related compensatory strategies to weekly goals. The iTEST coaching session materials are adapted from Cognitive Behavioral Social Skills Training (CBBST) [44] and Action-Based Cognitive Remediation [43]. The topic of each coaching session is outlined in Table 1.

**Table 1:** iTEST Coaching Sessions

Session Number	Topic	Skills Addressed
**1**	Introduction to iTEST, Introspective Accuracy, and Recovery Goals	•Psychoeducation about introspective accuracy •Discussion of meaningful recovery goals
**2**	Selecting Goal Steps and Applying Compensatory Skills in Plans	•Breaking recovery goals into goal steps •Applying compensatory cognitive skills to facilitate planning and goal step execution (e.g., calendaring, reminders)
**3**	Managing Unhelpful Expectations in Goal Steps	•Applying introspective accuracy to anticipation of engaging in goal steps •Assessing the probability and usefulness of expectations surrounding goal steps, and developing more balanced expectations •Integrating balanced expectations into reminders and calendaring
**4**	Sticking with the Facts During Goal Steps	•Working through examples of how under- and over-estimation of performance interfere with goal step completion •Strategies for collecting objective information during engagement in goal steps •Integrating balanced statements countering unhelpful appraisals during goal steps and incorporating into reminders and calendaring
**5**	Applying IA to Social Interactions	•Psychoeducation about the role of other people in attaining goal steps •Introspective accuracy in judgments about others’ intentions and feelings •Strategies for collecting objective information through communication
**6**	Learning from Experiences and Adjusting Goals	•Strategies for enhancing recall of the outcome of goal steps to prevent bias •Personalized approaches to adjusting based on outcome of goal steps and rewarding oneself for progress and integrating rewards into reminders •Review of iTEST sessions 1 through 6 and planning for booster sessions

***Coach training and fidelity*:** Coaches are bachelor’s or master’s level and have prior experience in clinical interviewing and/or intervention delivery in people with serious mental illnesses. Before the start of the study, coaches participated in four two-hour training workshops led by the PIs and an advanced doctoral student clinician, as well as training in safety planning. Coaches then conducted mock sessions which were video recorded for review. After the completion of training, coaches meet weekly for ongoing group supervision. Coaching sessions are audiotaped for supervision and fidelity ratings. Sessions are rated using an adapted version of the Cognitive Therapy Rating Scale for Psychosis (CTS-Psy) [45].

### Dosing framework

Because IA is a novel target, the most efficient dose is unknown. Our rationale for the dosing framework of iTEST is based on three parameters: (a) duration in weeks, (b) a number of trials, and (c) frequency of feedback. We set the starting duration of iTEST at 8 weeks with training 6 times per week (1x per day, ~15 min) consisting of 3 rounds of trials and feedback for both memory and emotion recognition tasks. As a comparison, an in-lab IA intervention in healthy adults conducted over 8 weeks included 2160 total training trials and 80 instances of IA feedback (in aggregate ~6 hours) [15]. iTEST at 8 weeks equates to 4320 training trials and 188 rounds of feedback (8–10 hours), which essentially doubles the dose of the previous in-person IA intervention [15]. We then extended this period to 16 weeks with additional evaluations at 12 and 16 weeks. We note that *the maximum* dose of training at 16 weeks is 75% of the mean training duration reported in a 2020 meta-analysis of available computerized cognitive training [46] and ~50% of the average duration of cognitive remediation programs [47].

### Assessments and measures

Table 2 details study assessments, which include an evaluation of current psychiatric symptoms (psychotic, mood, and suicidality), IA, and other factors that may influence treatment engagement such as motivation, sleep, and treatment expectancies. During the baseline visit, demographic and social contextual information (e.g., living situation) are collected and diagnostic evaluations are completed. Medication information, including sedation/sleepiness side effects, is collected via the Karolinska scale [48] at all assessment points. Two technology-based assessments, the Brief Assessment of Cognition in Schizophrenia-App Version (BACS) [49] and the Virtual Reality Functional Capacity Assessment Tool (VRFCAT) [50], are administered to assess global cognition and functional capacity, respectively, at baseline [51, 52]. All participants continue to receive their current mental health treatments with their previously established clinicians.

**Table 2:** Study Measures

Measures, Constructs, and Indicators		
**Construct**	**Measure**	**Operational Indicator**
**Sample Characterization/Exploratory Moderators**		
*Cognition*	BACS (Baseline only)	Total Score
*Functional Capacity*	VRFCAT (Baseline only)	Total Score
*Psychotic Symptoms*	PANSS Positive and Negative	Total Score
*Depressive Symptoms*	Calgary Depression Scale (CDSS)	Total Score
*Treatment Expectancies*	Credibility/Expectancy Questionnaire (CEQ)	Total Score
**Primary outcome: Trained Task-Based IA Target (R61,R33)**		
*Trained Task-based IA*	IA on METER and VLMT	Estimated vs Actual correct
**Primary outcome: Transfer of Training Target (R61, R33)**		
*Untrained Task-based IA*	Metacognitive WCST	Global resolution score
**Functional outcomes (Exploratory in R61; R33)**		
*Functional Outcome*	Informant-Rated SLOF	Total Score
**Secondary Outcomes**		
*Life space Mobility*	GPS	Daily distance from home
**Exploratory Mechanisms**		
*Strategy Use; Motivation and Defeatist Beliefs*	Compensatory Cognitive Strategies Scale; CAINS Motivation, Defeatist Performance Beliefs	Total scores
**Acceptability and Side Effects**		
*Intervention Satisfaction*	Client Satisfaction Questionnaire (CSQ)	Total Score
*User Engagement*	Trials and Coaching Sessions Completed	% of Total Possible
*Adverse Reactions*	Negative Effects Questionnaire (NEQ)	Total Score

*BACS* Brief Assessment of Cognition in Schizophrenia-App Version, *VRFCAT* Virtual Reality Functional Capacity Assessment Tool, *PANSS* Positive and Negative Syndrome Scale, *METER* Mobile Electronic Test of Emotional Recognition, *VLMT* Variable Difficulty List Memory Test, *WCST* Wisconsin Card Sorting Test, *SLOF* Specific Level of Functioning, *CAINS* Clinical Assessment Interview for Negative Symptoms.

***Measures for targets:*** In addition to feasibility and acceptability, the go/no go criteria to advance to a randomized clinical trial are improvements in IA on both trained and untrained tasks. For trained tasks, participants complete the METER and VLMT assessments on their smartphones during assessment weeks. Unlike the mobile training detailed above, the administration of METER and VLMT during assessment weeks does not include feedback about IA accuracy or task performance. The primary outcome is the averaged absolute discrepancy between each trial’s correct performance from that estimated by the participant, which is nested within sessions and weeks. We focused on trained tasks in IA as an outcome because a lack of improvement on these trained tasks could raise concerns about iTEST’s ability to modify IA and would suggest a need to revise the program.

An additional outcome is the transfer of training to an untrained task-based measure that is highly sensitive to IA: a modified version of the Meta-Cognitive Wisconsin Card Sorting Test (Metacognitive WCST) [53]. IA on this task is associated with functioning [4], and improvement on this task would support that iTEST leads to generalized improvement in IA since it addresses different cognitive domains than the training tasks. This Metacognitive WCST was developed by Koren (2006) and modified by our group [9, 53]. The primary outcome measure is the difference between momentary accuracy judgments and the correctness of individual sorts [9].

### Secondary measures

***Functional outcome:*** The Specific Level of Function (SLOF) scale is a 43-item interview used to measure functioning [54], and it will be administered to both participants and to their informants as an indicator of both real-world impact of iTEST (e.g., improvements in ratings) and of far transfer (e.g., the increased agreement between participants and informants). The scale assesses the participant’s current functioning integrated into a single higher-order factor addressing social, everyday, and vocational activities [55]. The SLOF emerged as the most reliable and externally valid scale-based measure of functioning in the team’s prior validation study [56]. Informant ratings on the SLOF were also found to be sensitive to short-term functional gains in a study conducted by our research group [57].

***Exploratory functional outcome*:** As an objective digital biomarker of mobility pertinent to functioning, we measure GPS life space with NeuroUX’s NeuroLogger app (https://www.getneuroux.com/neurologger). Participants can opt-out of this exploratory measure and still participate in the trial. As in our prior reports in which mobility was associated with community functioning [20, 58], outcomes are median daily distance traveled from home and percent of samples at home.

***Exploratory mechanistic targets linking change in IA to outcome*:** We will explore whether iTEST increases the use of compensatory strategies with the Compensatory Cognitive Strategies Scale [59], as well as motivation measured with Clinical Assessment Interview for Negative Symptoms (CAINS) Motivation and Pleasure (MAP) subscale [60]. We will also explore Defeatist Performance Beliefs (DPAS) [61], which predict effort [62] and may improve with more accurate self-awareness of abilities.

***Intervention satisfaction:*** Treatment expectations are measured at baseline using the Credibility/Expectancy Questionnaire (CEQ) [63]. An adapted version Client Satisfaction Questionnaire (CSQ) [64] keyed to both coaching sessions and mobile intervention components will be used. ***Symptom, safety, and side effect assessments:*** At each assessment timepoint, depression and psychotic symptoms are assessed with the Calgary Depression Scale for Schizophrenia (CDSS) [65] and Positive and Negative Syndrome Scale (PANSS) [31], respectively. We administer the Columbia Suicide Severity Rating Scale at each visit to assess suicide risk [66]. Since the change in IA could theoretically lead to adverse experiences in some individuals (e.g., greater awareness leading to depressed mood) [67], we will also administer the Negative Effects Questionnaire (NEQ) [68]. The CDSS, PANSS, CSSRS, and NEQ are administered at each follow-up session.

### Data safety monitoring

This study is supervised by an established clinical trials Data Safety Monitoring Board (DSMB) that consists of experts in clinical trials in serious mental illnesses and biostatistics, along with a patient advocate. The DSMB reviews the projects annually.

### Statistical approach

We will use Multilevel Structural Equation Modeling (MLSEM) with three levels to estimate changes in Introspective Accuracy (IA) across trials (level 1) and/or weeks (level 2) within persons (level 3). Given the relatively large number of random effects, we will use Bayesian estimation rather than maximum likelihood estimation for the analyses [69]. Although some task or ability domains will not be measured at every trial or every week, specifying trial as Level 1 (e.g., for IA on mobile task) and week as Level 2 (e.g., for Metacognitive WCST) will still enable us to estimate changes in IA for these domains. Our models assume the presence of missing data, and we will evaluate models under various assumptions of missingness (i.e., missing completely at random (MCAR), not at random (MNAR), or random (MAR)). We will investigate ceiling effects based on skewness and kurtosis and transform variables as needed.

***Sample size and power analyses:*** We used the Monte Carlo simulation capabilities of Mplus version 8.452 to perform our power analyses. To determine our levels of power to detect changes in IA for the list-learning task or emotion recognition tasks equivalent to *d* = |0.50|, we specified three-level models with 1000 replications each wherein trials (*t* = 18) were nested within weeks (8, 12, or 16) that were further nested within persons (*n* = 60 or 48 in the case of 20% attrition). This yielded strong power (i.e., > 0.90) across all combinations of sample size and week size. To determine our levels of power to detect transfer-of-training, we again specified three-level models with 1000 replications each wherein trials (*t* = 18) were nested within weeks (8, 12, or 16) that were further nested within persons (*n* = 60 or 48 in the case of 20% attrition). To determine our levels of power to detect exploratory predictors of changes in IA and their effects on functional outcomes, we modified the models such that predictors were regressed on changes in IA or functional outcomes were regressed on changes in IA. This yielded more-or-less adequate power ( ≈ 0.78–0.80) with 48 participants and fully adequate power (≈0.86–0.88) with 60 participants.

## Results

To inform intervention development for the iTEST software, we completed pre-implementation alpha testing with participants with psychotic disorders (*n* = 5 for 5 days, *n* = 10 for 3 weeks). This testing included 6 days per week of the revised VLMT and METER IA training tasks and was aimed at obtaining feedback on usability and adherence/engagement. Following alpha testing, we conducted iTEST beta testing with participants with psychotic disorders (UT at Dallas: *n* = 5; UC San Diego: *n* = 5) to assess the feasibility and usability of the mobile tasks on iTEST. Beta participants completed up to 6 days of tasks for one week, with flexibility to choose one rest day. Beta testers did not receive coaching. After 7 days, participants completed the System Usability Scale and provided qualitative feedback. Overall task completion rate was 96.5%. System Usability Scores ranged from 72.5–100 with an average score of 93.8 (SD 8.0), which is considered excellent and is well above the accepted benchmark score of 68 [70]. Qualitative ratings of iTEST indicated very little to no difficulty with the program and high overall satisfaction. Recruitment is in progress as of November 2023. Enrollment in the open trial stage is expected to be completed by April 2025.

## Discussion

Previous research indicates that introspective accuracy (IA) is impaired in people with psychotic disorders and is an independent predictor of functional outcomes [4–8]. However, no interventions have been developed and tested to improve IA. Experiments in healthy adults have indicated that IA can be modified and that improvements in one domain can be generalized to untrained tasks. Therefore, IA is a potential intervention target in psychotic disorders. Our trial represents a first step in testing whether IA is malleable in psychotic disorders, and whether improvements in trained domains generalize to untrained tasks. Further, we will assess whether coupling IA training with individualized coaching supports the translation of improved IA abilities to individually meaningful functional goal achievement. Although this trial is focused on a standalone intervention targeting IA, iTEST could be adapted to be coupled with cognitive training programs to expand training transfer and further impact functional outcomes.

Although several mobile interventions have been deployed in psychotic disorders [71], very few incorporate drill-and-practice mobile cognitive training. iTEST employs an app that engages with participants on a daily basis, coupled with coaching in applying IA to recovery goals consistent with elements of more effective cognitive remediation programs [34]. The mobile training experience includes design elements to increase engagement, such as streaks and digital art that grows with user engagement. This format may be useful for deploying cognition-targeted interventions in psychotic disorders.

Our operationalization of IA focuses on real-time, task-based judgments that can be directly compared to actual performance metrics. This differs from other approaches that measure IA through general self-ratings of abilities, confidence ratings, metacognitive questionnaires, or through comparisons between patient, family, and clinician ratings where determining accuracy becomes more complex. While each of these approaches is informative, in behavioral tasks we have an objective standard against which to measure self assessment accuracy, enabling precise quantification of the gap between perceived and actual performance. This methodological clarity is particularly valuable for intervention testing, as it allows us to track changes in accuracy over time and provide immediate, unambiguous feedback during training.

Further, while IA impairments often manifest as task-specific overconfidence, individuals with psychotic disorders may simultaneously underestimate their broader abilities. iTEST addresses this complexity through mobile training that targets task-specific IA with structured feedback, while coaching addresses broader self-evaluation patterns. The described study measures both aspects through performance tasks and the Defeatist Performance Beliefs Scale (DPAS).

Since iTEST is a new intervention, our trial is designed based on the NIMH’s experimental therapeutics framework, with a focus on evaluating the impact on both trained and untrained IA alongside feasibility and acceptability. The study design includes an embedded investigation of dosing, with evaluation of the go/no-go criteria assessed at 3 different follow-up time points (8, 12, and 16 weeks). If go/no go criteria are met, a planned next step is a larger efficacy-focused randomized controlled trial in which iTEST would be compared to a time-equivalent intervention employing both mobile assessments and coaching without training in IA. The earliest “dose” (8, 12, or 16 weeks) at which go/no go criteria are met would be selected. If go/no go criteria are met, a follow-on randomized control trial would evaluate whether iTEST improves functional outcomes to a greater extent an active comparator and whether a change in IA mediates the effect of iTEST on functional outcomes.

This pilot study of iTEST will provide important information on the malleability of IA in psychotic disorders and whether blended mobile cognition-targeted interventions might be a route to expanding access to treatments. Our findings may ultimately pave the way for more personalized and accessible cognitive remediation options, helping to bridge the gap between research and practical application in mental health treatment. Ultimately, this could help individuals with psychotic disorders achieve better functional outcomes and enhance their overall quality of life.

### Citation diversity statement

The authors have attested that they made efforts to be mindful of diversity in selecting the citations used in this article.

## Supplementary information

CONSORT Diagram

## References

[1] Wu EQ, Birnbaum HG, Shi L, Ball DE, Kessler RC, Moulis M, et al. The economic burden of schizophrenia in the United States in 2002. J Clin Psychiatry. 2005;66:1122–9. 16187769 10.4088/jcp.v66n0906

[2] Charlson FJ, Ferrari AJ, Santomauro DF, Diminic S, Stockings E, Scott JG, et al. Global epidemiology and burden of schizophrenia: findings from the global burden of disease study 2016. Schizophr Bull. 2018;44:1195–203. 29762765 10.1093/schbul/sby058 PMC6192504

[3] Rössler W, Salize HJ, van Os J, Riecher-Rössler A. Size of burden of schizophrenia and psychotic disorders. Eur Neuropsychopharmacol. 2005;15:399–409. 15925493 10.1016/j.euroneuro.2005.04.009

[4] Gould F, McGuire LS, Durand D, Sabbag S, Larrauri C, Patterson TL, et al. Self-assessment in schizophrenia: Accuracy of evaluation of cognition and everyday functioning. Neuropsychology. 2015;29:675–82. 25643212 10.1037/neu0000175 PMC4522405

[5] Silberstein J, Harvey PD. Impaired introspective accuracy in schizophrenia: an independent predictor of functional outcomes. Cogn Neuropsychiatry. 2019;24:28–39. 30477401 10.1080/13546805.2018.1549985 PMC6370513

[6] Durand D, Strassnig MT, Moore RC, Depp CA, Ackerman RA, Pinkham AE, et al. Self-reported social functioning and social cognition in schizophrenia and bipolar disorder: Using ecological momentary assessment to identify the origin of bias. Schizophr Res. 2021;230:17–23. 33667854 10.1016/j.schres.2021.02.011 PMC8222067

[7] Pinkham AE, Harvey PD, Penn DL. Social cognition psychometric evaluation: results of the final validation study. Schizophr Bull. 2018;44:737–48. 28981848 10.1093/schbul/sbx117 PMC6007629

[8] Harvey PD, Strassnig MT, Silberstein J. Prediction of disability in schizophrenia: Symptoms, cognition, and self-assessment. J Exp Psychopathol. 2019;10:2043808719865693.

[9] Tercero BA, Perez MM, Mohsin N, Moore RC, Depp C, Ackerman RA, et al. Using a meta-cognitive Wisconsin card sorting test to measure introspective accuracy in schizophrenia and bipolar disorder. J Psychiatr Res. In Press. 10.1016/j.jpsychires.2021.06.016 PMC8319124 34147931

[10] Koren D, Poyurovsky M, Seidman LJ, Goldsmith M, Wenger S, Klein EM. The neuropsychological basis of competence to consent in first-episode schizophrenia: a pilot metacognitive study. Biol psychiatry. 2005;57:609–16. 15780847 10.1016/j.biopsych.2004.11.029

[11] Altman RAE, Tan EJ, Rossell SL. Factors impacting access and engagement of cognitive remediation therapy for people with Schizophrenia: A systematic review. Can J Psychiatry. 2023;68:139–51. 36448242 10.1177/07067437221129073 PMC9974655

[12] Penney D, Sauve G, Mendelson D, Thibaudeau E, Moritz S, Lepage M. Immediate and sustained outcomes and moderators associated with metacognitive training for psychosis: a systematic review and meta-analysis. JAMA Psychiatry. 2022;79:417–29. 35320347 10.1001/jamapsychiatry.2022.0277 PMC8943641

[13] Mazancieux A, Fleming SM, Souchay C, Moulin CJA. Is there a G factor for metacognition? Correlations in retrospective metacognitive sensitivity across tasks. J Exp Psychol: Gen. 2020;149:1788–99. 32191079 10.1037/xge0000746 PMC7397761

[14] Schmidt C, Reyes G, Barrientos M, Langer ÁI, Sackur J. Meditation focused on self-observation of the body impairs metacognitive efficiency. Conscious Cognit. 2019;70:116–25. 30871785 10.1016/j.concog.2019.03.001

[15] Carpenter J, Sherman MT, Kievit RA, Seth AK, Lau H, Fleming SM. Domain-general enhancements of metacognitive ability through adaptive training. J Exp Psychol: Gen. 2019;148:51. 30596440 10.1037/xge0000505 PMC6390881

[16] Pratiwi I, Situmorang M, editors. Development of Contextual Based Modules to Improve Metacognition Abilityof Student. Proceeding International Conference of Science Education in Industrial Revolution 40; 2019.

[17] Cherrier S, Le Roux P-Y, Gerard F-M, Wattelez G, Galy O. Impact of a neuroscience intervention (NeuroStratE) on the school performance of high school students: Academic achievement, self-knowledge and autonomy through a metacognitive approach. Trends Neurosci Educ. 2020;18:100125. 32085909 10.1016/j.tine.2020.100125

[18] Kanar AM, Bouckenooghe D. Prompting metacognition during a job search: evidence from a randomized controlled trial with university job seekers. Appl Psychol. 2021;70:955--85.

[19] Kleitman S, Narciss S. Introduction to the special Issue “applied metacognition: real-world applications beyond learning. Metacognition Learn. 2019;14:335–42.

[20] Parrish EM, Depp CA, Moore RC, Harvey PD, Mikhael T, Holden J, et al. Emotional determinants of life-space through GPS and ecological momentary assessment in schizophrenia: What gets people out of the house? Schizophrenia Res. 2020;224:67–73. 10.1016/j.schres.2020.10.002 PMC13085614 33289659

[21] Depp CA, Kamarsu S, Filip TF, Parrish EM, Harvey PD, Granholm EL, et al. Ecological momentary facial emotion recognition in psychotic disorders. Psychol Med. 2021:1–9. 10.1017/S0033291720004419 PMC8621678 33431072

[22] Morgan O, Strassnig MT, Moore RC, Depp CA, Ackerman RA, Pinkham AE, et al. Accuracy of immediate self-assessment of neurocognitive test performance: Associations with psychiatric diagnosis and longitudinal psychotic symptoms. J Psychiatr Res. 2022;156:594–601. 36372002 10.1016/j.jpsychires.2022.10.069 PMC9899150

[23] Green MF, Kern RS, Braff DL, Mintz J. Neurocognitive deficits and functional outcome in schizophrenia: are we measuring the “right stuff”? Schizophr Bull. 2000;26:119–36. 10755673 10.1093/oxfordjournals.schbul.a033430

[24] Pinkham AE, Penn DL, Perkins DO, Lieberman J. Implications for the neural basis of social cognition for the study of schizophrenia. Am J Psychiatry. 2003;160:815–24. 12727681 10.1176/appi.ajp.160.5.815

[25] Fervaha G, Foussias G, Takeuchi H, Agid O, Remington G. Motivational deficits in major depressive disorder: Cross-sectional and longitudinal relationships with functional impairment and subjective well-being. Compr Psychiatry. 2016;66:31–8. 26995233 10.1016/j.comppsych.2015.12.004

[26] Depp CA, Kamarsu S, Filip TF, Parrish EM, Harvey PD, Granholm EL, et al. Ecological momentary facial emotion recognition in psychotic disorders. Psychol Med. 2022;52:2531–9. 33431072 10.1017/S0033291720004419 PMC8621678

[27] Parrish EM, Kamarsu S, Harvey PD, Pinkham A, Depp CA, Moore RC. Remote ecological momentary testing of learning and memory in adults with serious mental illness. Schizophr Bull. 2021;47:740–50. 33219382 10.1093/schbul/sbaa172 PMC8084440

[28] Cobb N, Witte E, Cervone M, Kirby A, MacFadden D, Nadler L, et al. The SMART IRB platform: A national resource for IRB review for multisite studies. J Clin Transl Sci. 2019;3:129–39. 31660237 10.1017/cts.2019.394 PMC6798516

[29] Jeste DV, Palmer BW, Appelbaum PS, Golshan S, Glorioso D, Dunn LB, et al. A new brief instrument for assessing decisional capacity for clinical research. Arch Gen psychiatry. 2007;64:966–74. 17679641 10.1001/archpsyc.64.8.966

[30] Wilkinson GS, Robertson GJ, Psychological Assessment Resources I. WRAT 4 : wide range achievement test ; professional manual. Lutz, FL: Psychological Assessment Resources, Inc.; 2006.

[31] Kay SR, Fiszbein A, Opler LA. The positive and negative syndrome scale (PANSS) for schizophrenia. Schizophr Bull. 1987;13:261–76. 3616518 10.1093/schbul/13.2.261

[32] Tercero BA, Perez MM, Mohsin N, Moore RC, Depp CA, Ackerman RA, et al. Using a Meta-cognitive Wisconsin Card Sorting Test to measure introspective accuracy and biases in schizophrenia and bipolar disorder. J Psychiatr Res. 2021;140:436–42. 34147931 10.1016/j.jpsychires.2021.06.016 PMC8319124

[33] Badal VD, Depp CA, Moore R, Ackerman RA, Harvey PD, Pinkham A. Confidence, accuracy judgments and feedback in schizophrenia and bipolar disorder: a time series network analysis. Psychol Med. 2023;53:4200–9. 10.1017/S0033291722000939 35478065

[34] Vita A, Barlati S, Ceraso A, Nibbio G, Ariu C, Deste G, et al. Effectiveness, core elements, and moderators of response of cognitive remediation for schizophrenia: a systematic review and meta-analysis of randomized clinical trials. JAMA Psychiatry. 2021;78:848–58. 33877289 10.1001/jamapsychiatry.2021.0620 PMC8058696

[35] Parrish EM, Kamarsu S, Harvey PD, Pinkham A, Depp CA, Moore RC. Remote ecological momentary testing of learning and memory in adults with serious mental illness. Schizophr Bull. 2021;47:740--50.. 10.1093/schbul/sbaa172 PMC8084440 33219382

[36] Dark FL, Amado I, Erlich MD, Ikezawa S. International experience of implementing cognitive remediation for people with psychotic disorders. Schizophr Bull. 2024;50:1017--27. 10.1093/schbul/sbae071 PMC11349011 38758086

[37] Domhardt M, Geßlein H, von Rezori RE, Baumeister H. Internet- and mobile-based interventions for anxiety disorders: A meta-analytic review of intervention components. Depress Anxiety. 2019;36:213–24. 30450811 10.1002/da.22860

[38] Qin Y, Wang X, Namkoong K. A meta-analysis of the overall effect of mHealth physical activity interventions for weight loss and the moderating effect of behavioral change theories, techniques, and mobile technologies. Mob Media Commun. 2022;10:337–59.

[39] Granholm E, Holden J, Dwyer K, Mikhael T, Link P, Depp C. Mobile-assisted cognitive behavioral therapy for negative symptoms: open single-arm trial with schizophrenia patients. JMIR Ment Health. 2020;7:e24406. 33258792 10.2196/24406 PMC7738249

[40] Depp C, Ehret B, Villa J, Perivoliotis D, Granholm E. A brief mobile-augmented suicide prevention intervention for people with psychotic disorders in transition from acute to ongoing care: protocol for a pilot trial. JMIR Res Protoc. 2021;10:e14378. 33555265 10.2196/14378 PMC7899804

[41] Depp CA, Ceglowski J, Wang VC, Yaghouti F, Mausbach BT, Thompson WK, et al. Augmenting psychoeducation with a mobile intervention for bipolar disorder: a randomized controlled trial. J Affect Disord. 2015;174:23–30. 25479050 10.1016/j.jad.2014.10.053 PMC4339469

[42] Depp CA, Perivoliotis D, Holden J, Dorr J, Granholm EL. Single-session mobile-augmented intervention in serious mental illness: a three-arm randomized controlled trial. Schizophr Bull. 2019;45:752–62. 30281086 10.1093/schbul/sby135 PMC6581143

[43] Bowie CR, Grossman M, Gupta M, Holshausen K, Best MW. Action-based cognitive remediation for individuals with serious mental illnesses: Effects of real-world simulations and goal setting on functional and vocational outcomes. Psychiatr Rehabil J. 2017;40:53. 27100095 10.1037/prj0000189

[44] Granholm E, Holden J, Link PC, McQuaid JR. Randomized clinical trial of cognitive behavioral social skills training for schizophrenia: improvement in functioning and experiential negative symptoms. J Consult Clin Psychol. 2014;82:1173. 24911420 10.1037/a0037098 PMC4244255

[45] Haddock G, Devane S, Bradshaw T, McGovern J, Tarrier N, Kinderman P, et al. An investigation into the psychometric properties of the Cognitive Therapy Scale for Psychosis (Cts-Psy). Behav Cogn Psychother. 2001;29:221–33.

[46] Bonnechère B, Langley C, Sahakian BJ. The use of commercial computerised cognitive games in older adults: a meta-analysis. Sci Rep. 2020;10:15276. 32943742 10.1038/s41598-020-72281-3 PMC7498601

[47] Cella M, Preti A, Edwards C, Dow T, Wykes T. Cognitive remediation for negative symptoms of schizophrenia: a network meta-analysis. Clin Psychol Rev. 2017;52:43–51. 27930934 10.1016/j.cpr.2016.11.009

[48] Shahid A, Wilkinson K, Marcu S, Shapiro CM Karolinska sleepiness scale (KSS). STOP, THAT and one hundred other sleep scales: Springer; 2011. 209-10.

[49] Keefe RS, Harvey PD, Goldberg TE, Gold JM, Walker TM, Kennel C, et al. Norms and standardization of the Brief Assessment of Cognition in Schizophrenia (BACS). Schizophr Res. 2008;102:108–15. 18495435 10.1016/j.schres.2008.03.024

[50] Keefe RSE, Davis VG, Atkins AS, Vaughan A, Patterson T, Narasimhan M, et al. Validation of a computerized test of functional capacity. Schizophr Res. 2016;175:90–6. 27091656 10.1016/j.schres.2016.03.038 PMC4958510

[51] Nuechterlein KH, Green MF, Kern RS, Baade LE, Barch DM, Cohen JD, et al. The MATRICS Consensus Cognitive Battery, part 1: test selection, reliability, and validity. Am J Psychiatry. 2008;165:203–13. 18172019 10.1176/appi.ajp.2007.07010042

[52] Patterson TL, Goldman S, McKibbin CL, Hughs T, Jeste DV. UCSD Performance-Based Skills Assessment: development of a new measure of everyday functioning for severely mentally ill adults. Schizophr Bull. 2001;27:235–45. 11354591 10.1093/oxfordjournals.schbul.a006870

[53] Koren D, Seidman LJ, Goldsmith M, Harvey PD. Real-world cognitive—and metacognitive—dysfunction in schizophrenia: a new approach for measuring (and remediating) more “right stuff. Schizophr Bull. 2006;32:310–26. 16397202 10.1093/schbul/sbj035 PMC2632222

[54] Schneider LC, Struening EL, editors. SLOF: a behavioral rating scale for assessing the mentally ill. Social Work Research and Abstracts; 1983: Oxford University Press. 10.1093/swra/19.3.9 10264257

[55] Bowie CR, Best MW, Depp C, Mausbach BT, Patterson TL, Pulver AE, et al. Cognitive and functional deficits in bipolar disorder and schizophrenia as a function of the presence and history of psychosis. Bipolar Disord. 2018;20:604–13. 29777563 10.1111/bdi.12654

[56] Harvey PD, Raykov T, Twamley EW, Vella L, Heaton RK, Patterson TL. Validating the measurement of real-world functional outcomes: phase I results of the VALERO study. Am J Psychiatry. 2011;168:1195–201. 21572166 10.1176/appi.ajp.2011.10121723 PMC3670945

[57] Bowie CR, Grossman M, Gupta M, Oyewumi LK, Harvey PD. Cognitive remediation in schizophrenia: efficacy and effectiveness in patients with early versus long-term course of illness. Early Interven Psychiatry. 2014;8:32–8. 10.1111/eip.12029 23343011

[58] Depp CA, Bashem J, Moore RC, Holden JL, Mikhael T, Swendsen J, et al. GPS mobility as a digital biomarker of negative symptoms in schizophrenia: a case control study. NPJ Digit Med. 2019;2:1–7. 31728415 10.1038/s41746-019-0182-1 PMC6841669

[59] Becker H, Stuifbergen AK, Henneghan A, Morrison J, Seo EJ, Zhang W. An initial investigation of the reliability and validity of the Compensatory Cognitive Strategies Scale. Neuropsychol Rehabil. 2019;29:739–53. 28552019 10.1080/09602011.2017.1329154 PMC5708149

[60] Kring AM, Gur RE, Blanchard JJ, Horan WP, Reise SP. The clinical assessment interview for negative symptoms (CAINS): final development and validation. Am J Psychiatry. 2013;170:165–72. 23377637 10.1176/appi.ajp.2012.12010109 PMC3785242

[61] Weissman AN, Beck AT Development and validation of the Dysfunctional Attitude Scale: A preliminary investigation. 1978.

[62] Reddy LF, Horan WP, Barch DM, Buchanan RW, Gold JM, Marder SR, et al. Understanding the association between negative symptoms and performance on effort-based decision-making tasks: the importance of defeatist performance beliefs. Schizophr Bull. 2018;44:1217–26. 29140501 10.1093/schbul/sbx156 PMC6192468

[63] Devilly GJ, Borkovec TD. Psychometric properties of the credibility/expectancy questionnaire. J Behav Ther Exp Psychiatry. 2000;31:73–86. 11132119 10.1016/s0005-7916(00)00012-4

[64] Larsen DL, Attkisson CC, Hargreaves WA, Nguyen TD. Assessment of client/patient satisfaction: Development of a general scale. Evaluation and Program Planning. 1997;2:197--207. 10.1016/0149-7189(79)90094-6 10245370

[65] Addington J, Shah H, Liu L, Addington D. Reliability and validity of the Calgary Depression Scale for Schizophrenia (CDSS) in youth at clinical high risk for psychosis. Schizophr Res. 2014;153:64–7. 24439270 10.1016/j.schres.2013.12.014 PMC3982913

[66] Posner K, Brent D, Lucas C, Gould M, Stanley B, Brown G, et al. Columbia-suicide severity rating scale (C-SSRS). New York, NY: Columbia University Medical Center. 2008.

[67] Murri MB, Respino M, Innamorati M, Cervetti A, Calcagno P, Pompili M, et al. Is good insight associated with depression among patients with schizophrenia? Systematic review and meta-analysis. Schizophr Res. 2015;162:234--47. 10.1016/j.schres.2015.01.003 25631453

[68] Rozental A, Kottorp A, Boettcher J, Andersson G, Carlbring P. Negative effects of psychological treatments: an exploratory factor analysis of the negative effects questionnaire for monitoring and reporting adverse and unwanted events. PLoS One. 2016;11:e0157503. 27331907 10.1371/journal.pone.0157503 PMC4917117

[69] Hamaker EL, Klugkist I. Bayesian estimation of multilevel models. Handbook for advanced multilevel analysis. European Association for Methodology series. New York, NY, US: Routledge/Taylor & Francis Group; 2011;137–61.

[70] Hyzy M, Bond R, Mulvenna M, Bai L, Dix A, Leigh S, et al. System usability scale benchmarking for digital health apps: meta-analysis. JMIR mHealth and uHealth. 2022;10:e37290. 10.2196/37290 PMC9437782 35980732

[71] Bell IH, Lim MH, Rossell SL, Thomas N. Ecological Momentary Assessment and Intervention in the Treatment of Psychotic Disorders: A Systematic Review. Psychiatric Services. 03 July 2017;68. 10.1176/appi.ps.201600523 28669284

