# Supplementary material for: Protocol for evaluation of iTEST, a novel blended intervention to enhance introspective accuracy in psychotic disorders
Source: NPP Digit Psychiatry Neurosci. 2025 Feb 14;3:5. doi: 10.1038/s44277-024-00024-7 (PMC11825358; doi:10.1038/s44277-024-00024-7)
Supplement: Supplementary file 1 — CONSORT Diagram [file 44277_2024_24_MOESM1_ESM.pptx]

## Slide 1
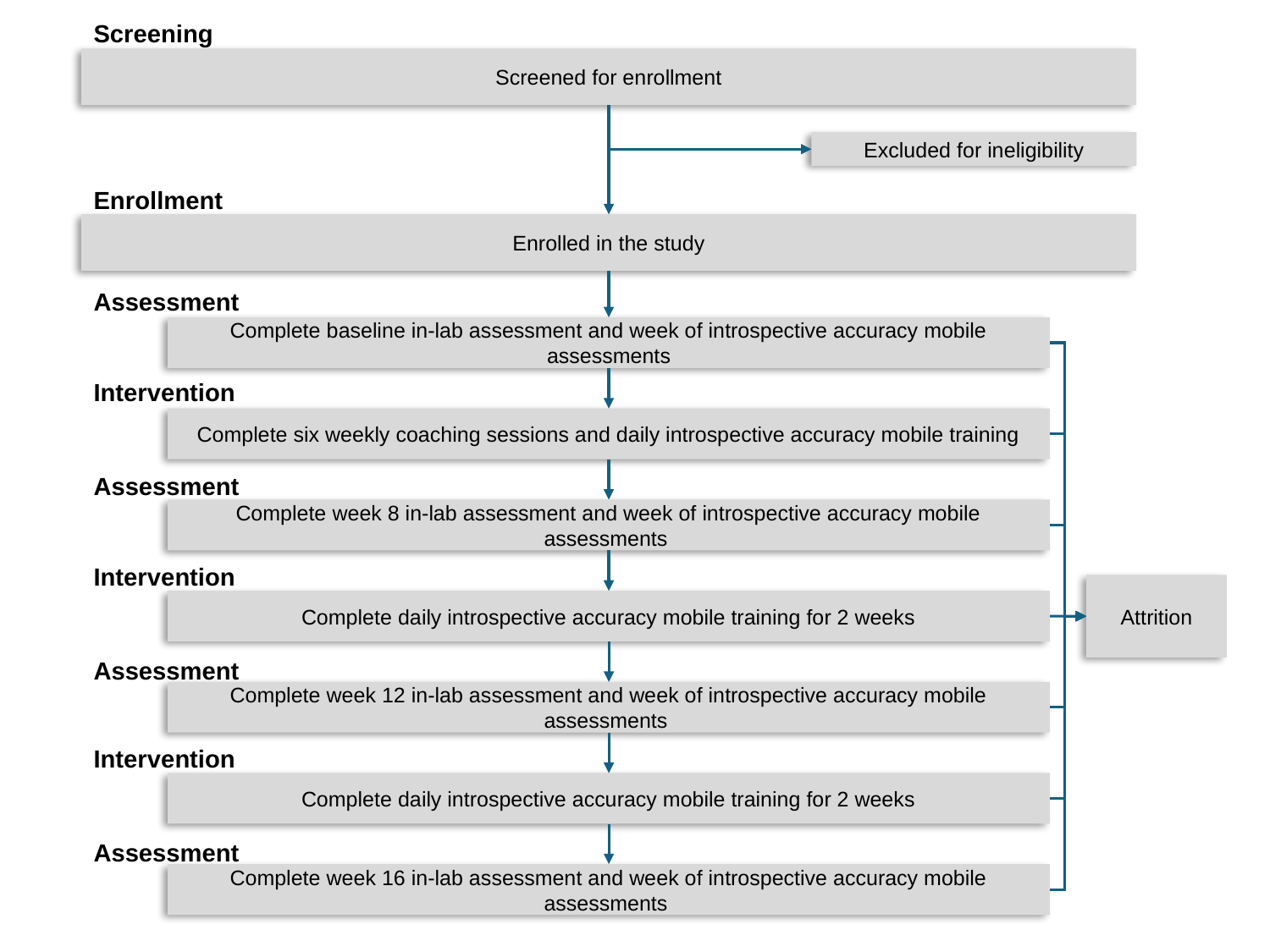

Screening
Screened for enrollment
Excluded for ineligibility
Enrollment
Enrolled in the study
Assessment
Complete baseline in-lab assessment and week of introspective accuracy mobile assessments
Intervention
Complete six weekly coaching sessions and daily introspective accuracy mobile training
Assessment
Complete week 8 in-lab assessment and week of introspective accuracy mobile assessments
Intervention
Attrition
Complete daily introspective accuracy mobile training for 2 weeks
Assessment
Complete week 12 in-lab assessment and week of introspective accuracy mobile assessments
Intervention
Complete daily introspective accuracy mobile training for 2 weeks
Assessment
Complete week 16 in-lab assessment and week of introspective accuracy mobile assessments
